# Risk Recognition and Phenotyping in Cardiac and Vascular Diseases

**DOI:** 10.3390/biomedicines14071583

**Published:** 2026-07-15

**Authors:** Ivano Barnaba, Francesco Massari, Pietro Scicchitano

**Affiliations:** 1 Cardiology Section, University of Bari, 70124 Bari, BA, Italy; ivanobarnaba@gmail.com; 2 Cardiology Section, Hospital “F. Perinei” ASL BA, 70022 Altamura, BA, Italy; franco_massari@libero.it

Cardiac and vascular diseases can no longer be interpreted by clinicians as rigid categories or fixed diagnoses; they are dynamic processes sustained by myocardial and vascular remodelling, endothelial dysfunction, inflammation, metabolic alterations, and thrombosis. These mechanisms interact each other at multiple levels and potentiate the risk of adverse events [1].

Studies in the field of cardiovascular diseases remain essential, primarily due to their epidemiological burden as the leading cause of death worldwide and status as one of the most relevant components of the global impact of disease. In 2023, they caused 19.2 million deaths and approximately 437 million disability-adjusted life years (DALYs) [2]. Arterial hypertension [3], metabolic disorders, and heart failure are increasingly contributing to these epidemiological data, thus forcing clinicians to adopt a truly multidisciplinary approach which should be oriented toward both research and patient management [2].

The third edition of the Special Issue “Cardiac and Vascular Diseases: Pathogenesis, Pharmacological Treatments, Advances in Therapies” brings together six contributions that span this broad spectrum, from emergency clinical care to molecular pharmacology. We present them along a narrative thread that starts at the patient’s bedside and reaches the laboratory.

In the first study, Scicchitano and colleagues evaluated the prognostic impact of non-invasive ventilation (NIV) in acute heart failure. This was a retrospective study which involved 200 patients admitted to a cardiac intensive care unit. The authors showed that patients who received NIV had a more severe clinical profile, longer hospital stays, and higher mortality, both in hospital and at 180-day follow-up. After adjustment for age, BNP, CRP, arterial blood gas parameters, renal function, and LVEF, NIV remained an independent predictor of six-month mortality (HR 1.61) [4]. This finding does not necessarily imply a causal relationship: NIV should not be interpreted per se as a cause of worse prognosis, but rather as a clinical signal—for the physician—of a higher frailty patient phenotype, often characterized by greater congestion. The European Society of Cardiology (ESC) guidelines also support its use in patients with acute heart failure and respiratory distress [5,6].

Zlibut and colleagues shift the focus to hypertrophic cardiomyopathy (HCM), a condition in which preserved ejection fraction can often mask myocardial dysfunction that is already underway and not always detectable using conventional parameters of ventricular function [7]. This was a prospective case–control study which enrolled 150 patients with HCM and 100 healthy controls matched for age and sex. Standardized CMR protocols and feature-tracking analysis were adopted. Despite a preserved ejection fraction (EF) condition, patients with HCM showed significantly worse values than those of the control group, with reductions in longitudinal, circumferential, and radial strain (GLS −16% vs. −20%; GCS −18% vs. −21%; GRS 29% vs. 38%) and global left ventricular torsion (1.27°/cm vs. 1.95°/cm) [7].

These alterations were also observed in subjects without late gadolinium enhancement, suggesting the presence of early functional abnormalities not yet evident when using some of the structural markers most commonly applied for HCM. Ventricular torsion appears to be a promising marker of subclinical dysfunction, and could also be used to identify early myocardial damage. However, the proposed cut-off will need to be confirmed in independent cohorts before being incorporated into routine clinical decision-making [7].

Csoma and colleagues evaluated exhaled nitric oxide parameters, particularly alveolar NO concentration (CANO) and bronchial NO flux (JawNO), in patients with pre-capillary pulmonary hypertension (PH) [8]. This was an observational study including 52 patients with pre-capillary PH and a control group of 27 subjects. CANO was higher in patients with pre-capillary PH than in controls and was inversely correlated with the diffusing capacity of the lung for carbon monoxide (DLCO), suggesting a link between alveolar NO and alterations of the alveolar–capillary compartment [8]. The authors propose, for the first time, the hypothesis that JawNO may serve as a non-invasive marker of clinical and functional response, with potential hemodynamic relevance, although prospective and multicenter confirmation remains necessary.

Cojocaru and colleagues address the role of thrombolysis in venous thromboembolism (VTE) during the post-COVID phase. Compared with the other patients, those who underwent thrombolysis had lower TAPSE, reduced peripheral oxygen saturation, and a smaller left ventricular end-diastolic diameter. Moreover, each 1 mm decrease in TAPSE and 1– decrease in peripheral oxygen saturation (SpO_2_) was related to an increased probability of thrombolysis, with odds ratios of 1.58 and 1.34, respectively [9]. This suggests that a reduction in these two parameters can be used to identify patients who are more likely to be at risk or hemodynamically compromised, and therefore more likely to be considered for an aggressive therapy such as thrombolysis. These findings demonstrate a clinical–echocardiographic triad that can be used at the patient’s bedside to guide early risk stratification, especially when advanced imaging or invasive monitoring are not immediately available. The interpretation of this study is supported by the pathophysiological rationale: hypercoagulability and endotheliopathy may persist after acute SARS-CoV-2 infection [10]; right ventricular dysfunction has been frequently described in patients with COVID-19, affecting approximately one in five patients [11]; and neutrophil extracellular traps (NETs) appear to contribute to mechanisms of microvascular thrombosis [12].

On the interventional front, Bil and colleagues compared dedicated bifurcation stents with conventional drug-eluting stents. They observed no significant differences in all-cause mortality, myocardial infarction, or target lesion revascularization at one- and four-year follow-ups, even in the subgroups with left main involvement [13]. Despite innovations, technologies should be used with caution in order to avoid alterations.

This collection closes with a translational study that shifts the focus from the clinical and interventional dimensions to computational pharmacology. This is an in silico study in which Abdelsayed and Boulaamane, through computational simulations on 3957 Food and Drug Administration-approved molecules, sought to identify molecules capable of simultaneously targeting three acute myeloid leukemia (AML) targets, namely LSD1, BCL-2, and mutant IDH1, particularly R132H. The authors identified three compounds, including belumosudil and elraglusib, which showed good simulated affinity for all three targets and favorable pharmacokinetic profiles, although they have not yet been validated in experimental AML models [14]. Therefore, computational screening, drug repurposing, and polypharmacology may become useful tools for the potential development of new drugs and for rethinking therapeutic strategies, especially in those settings marked by biological heterogeneity and treatment resistance [15].

Taken together, these six studies reveal a common direction: cardiovascular and biomedical innovation does not arise solely from new drugs or devices, but from the ability to better recognize risk, define disease phenotypes with greater precision, and select diagnostic and therapeutic strategies in a more rational way. This Special Issue clearly demonstrates that diagnosing a disease is not enough: it is necessary to understand which patients are more fragile or may progress toward instability, and which subclinical signals can be detected early with more sensitive tools, so as to make prevention and treatment more targeted.

At the same time, innovation should not be interpreted as a solution that can be applied indiscriminately to all patients, but rather as a tool to be adapted to the individual patient and their clinical, biological, and anatomical profile. Medicine is moving toward an increasingly personalized approach, supported by emerging tools such as computational simulations, which may suggest new drugs, therapeutic strategies, and hypotheses that might not emerge through traditional approaches. We hope that the studies collected in this Special Issue will foster new research questions and increasingly precise, timely, and personalized clinical practice.
